# Comparing the Immune Response to PEEK as an Implant Material with and without P-15 Peptide as Bone Graft Material in a Rabbit Long Bone Model

**DOI:** 10.3390/bioengineering11090898

**Published:** 2024-09-06

**Authors:** Boyle C. Cheng, Isaac R. Swink, Cooper T. Cheng, Owen G. Corcoran, Vicki Z. Wang, Edward J. McClain, Praveer S. Vyas, Izzy Owen, Chen Xu, Daniel T. Altman, Alexander K. Yu

**Affiliations:** 1 Neuroscience Institute, Allegheny General Hospital, Allegheny Health Network, Pittsburgh, PA 15212, USA; isaac.swink@ahn.org (I.R.S.); cooper.cheng@ahn.org (C.T.C.); owen.corcoran@ahn.org (O.G.C.); vicki.wang@ahn.org (V.Z.W.); praveer.vyas@ahn.org (P.S.V.); owenizzy12@gmail.com (I.O.); 2 Department of Neurosurgery, Allegheny General Hospital, Allegheny Health Network, Pittsburgh, PA 15212, USA; chen.xu@ahn.org (C.X.); alexander.yu@ahn.org (A.K.Y.); 3 Department of Orthopedic Surgery, Allegheny General Hospital, Allegheny Health Network, Pittsburgh, PA 15212, USA; daniel.altman@ahn.org

**Keywords:** P-15, PEEK, bone graft, bone regeneration, osteogenesis, immune response, inflammation, long bone, rabbits

## Abstract

P-15 is a 15-amino-acid-long biomimetic peptide widely demonstrated to enhance osteogenesis in vivo. Despite the prevalence of polyether-ether-ketone (PEEK) in interbody device manufacturing, a growing body of evidence suggests it may produce an unfavorable immune response. The purpose of this preliminary study was to characterize the immune response and new bone growth surrounding PEEK implants with and without a P-15 peptide-based osteobiologic. A bilateral femoral defect model was conducted using New Zealand white rabbits. A total of 17 test subjects received one implant in each distal femur, either with or without bone graft material. Animals were allowed to survive to 4 or 8 weeks, at which time the femurs were collected and subjected to micro-computer tomography (microCT) or cytokine analysis. MicroCT analysis included the quantification of bone growth and density surrounding each implant. The cytokine analysis of periprosthetic tissue homogenates included the quantification of interleukins (ILs) and TNF-α expression via ELISA kits. Improvements in bone volume were observed in the P-15 cohort for the regions of interest, 500–136 and 136–0 µm from the implant surface, at 8 weeks post-op. Concentrations of IL-1β, IL-4, and IL-6 cytokines were significantly higher in the P-15 cohort compared to the PEEK cohort at the 4-week timepoint. Significant reductions in the concentrations of IL-4 and IL-6 cytokines from the 4- to 8-week cohort were observed in the P-15 cohort only. The P-15 peptide has the potential to modulate the immune response to implanted materials. We observed improvements in bone growth and a more active micro-environment in the P-15 cohort relative to the PEEK control. This may indicate an earlier transition from the inflammatory to remodeling phase of healing.

## 1. Introduction

Following an injury or surgery to the bone, several phases of healing have been identified, including inflammation, repair, and remodeling [1,2]. During the inflammatory phase, cells from the innate immune system (neutrophils, monocytes, and stem cells) are recruited to the site of injury and secrete pro-inflammatory cytokines. Following inflammation, there is a shift from pro-inflammatory signaling to anti-inflammatory signals. A sustained pro-inflammatory state can disrupt healing and result in a non-union, or in certain cases, implant failure. In the repair phase, anti-inflammatory cytokines are produced, and native cell types are recruited to the wound [3]. These cells proliferate and differentiate to increase bony deposition to heal the wound space. During the remodeling phase, pro-inflammatory signaling is increased, resulting in a balance of resorption by osteoclasts and lamellar bone deposition by osteoblasts [4].

Understanding and controlling this foreign body reaction is critical to the success of orthopedic implants given it is the leading cause of aseptic loosening [5]. A recent survey shows that polyether-ether-ketone (PEEK) is one of the most-used materials in these procedures, with upwards of 84% of all cervical interbody devices being manufactured with this material [6]. Despite the prevalence of PEEK, there is a growing body of evidence that it may produce an unfavorable immune response, leading to fibrous encapsulation or adipose tissue formation rather than osseointegration [7,8].

An alternate common implant material is titanium, to which PEEK is often qualitatively compared to. Results of in vitro investigations show an increased expression of inflammatory cytokines such as IL-1β, IL-6, IL-8, and TNF-α in cells cultured on PEEK as compared to titanium. This upregulation may be a precursor to fibrogenesis surrounding PEEK implants [9]. However, an important factor in the choice of implant material is the elastic modulus because mismatches in elastic moduli can cause poor surgical outcomes such as pseudoarthrosis, adjacent segment disease, and implant subsidence. PEEK, as opposed to titanium, remains a preferred interbody device material since its elastic modulus is more akin to that of bone [10]. An additional consideration is that the radiodensity of titanium implants can cause the visual indicators of poor surgical outcomes to be hidden under X-ray examination. The radiolucency of PEEK instead allows for an unobstructed view of the implant site [11]. For these reasons, research into improving the immune response to PEEK remains a subject of interest.

A retrospective review conducted by Krause et al. showed that of the 127 patients who underwent anterior discectomy and fusion, 52% of the patients treated with PEEK implants (29/56) exhibited radiographic evidence of pseudoarthrosis at the 1-year follow-up, as compared to 10% of the patients treated with structural allograft (7/71) [12]. This is in stark contrast to a 2016 study that reported a 95% fusion rate at the 1-year follow-up in PEEK cages filled with a bone graft substitute (21/22) [13]. While bone graft substitutes are only one factor which may influence the success of spinal fusion procedures, this indicates bone graft substitutes may be able to modulate the immune response to PEEK implants.

Although BMP-2 is the most common osteoinductive growth factor used in the field of bone repair, the supraphysiologic concentrations required for its function result in a variety of side effects, including inflammatory soft tissue swelling, fibrotic encapsulation, cyst-like bone void formation (fatty bone), ectopic bone formation, and osteoclast activation with increased resorption [14,15]. The risks inherent to BMP-2 treatment necessitate research into P-15 as an alternate bone graft material for use with interbody devices. P-15 is a well-studied 15-amino-acid-long peptide which mimics the cell-binding domain of human type I collagen and has shown success in promoting osteoblast adhesion and differentiation [16]. Several studies have indicated that P-15 promotes enhanced cell attachment, proliferation, and differentiation. A mechanism has been reported based on the abundance of these studies, which occurs through three phases including attachment, activation, and amplification. These three phases are complementary to the archetypical bone healing phases of inflammation, repair, and remodeling [17]. We hypothesize in this preliminary study that while P-15 promotes success for the osteogenic lineage, it also modulates the immune cell response to promote a pro-healing environment when used in conjunction with PEEK implants.

## 2. Materials and Methods

### 2.1. Preparation of Bone Dowels

**Figure 1:**
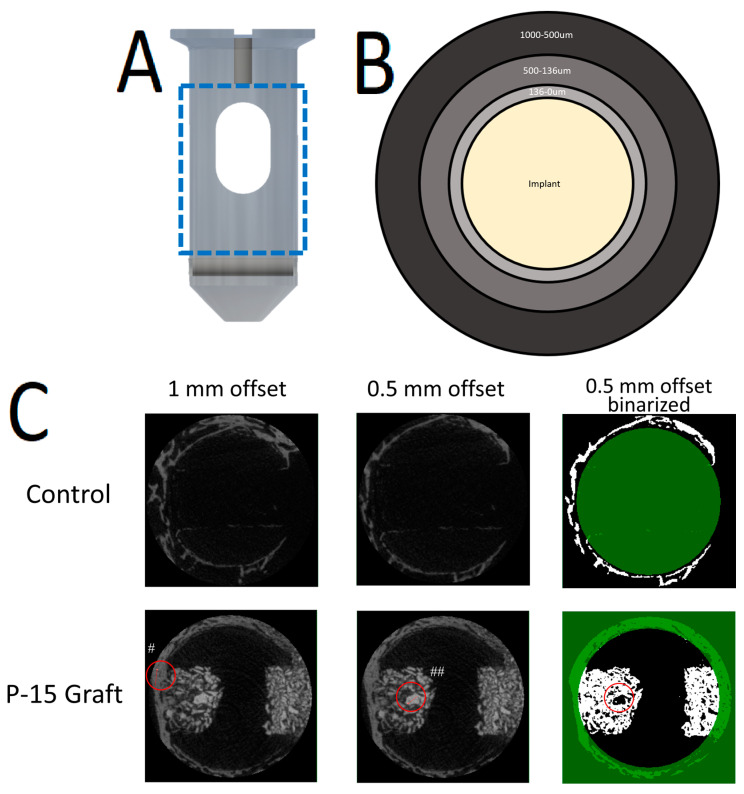
Illustration of microCT methods. (**A**) PEEK bone dowel. Dashed blue line illustrates where the analysis was performed along the central aspect of the implant. (**B**) Locations of each of the 3 regions of interest (ROIs). (**C**) Micro-computer tomography (microCT) analysis. Red circles illustrate areas where bone density was investigated to determine the thresholding window. # indicates bone with a relative grayscale value (GV) of ~100. ## indicates a calcium phosphate particle with a GV of ~150. The thresholding window was set to 45—123 to remove as much residual calcium phosphate as possible.

Implants (6 × 16 mm, Allegheny Performance Plastics, Pittsburgh, PA, USA) with a central canal were machined from medical-grade Ketaspire KT-820 NT rod stock (PEEK). Two tantalum pins were added to the dowels to allow for determination of implant orientation during analysis and aid in appropriate implant placement (Figure 1A).

### 2.2. Animal Model

**Figure 2:**
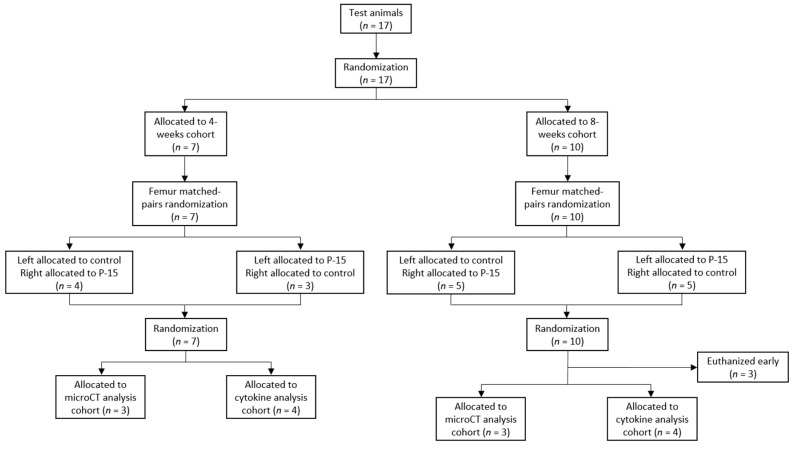
Randomization flowchart of animals to timepoint and treatment cohorts.

A bilateral bone defect model was used to compare the immune response between PEEK dowels with and without P-15 bone graft. A total of 17 skeletally-mature male New Zealand white rabbits (age: 6 months; body weight: 3–4 kg) were each implanted with one dowel in each distal femur to allow for a paired analysis. All animal care and experimental protocols were reviewed and approved by the Allegheny Health Network Institutional Animal Care and Use Committee (IACUC 1083). The graft product used in all animals was a combination product consisting of P-15 peptide-coated anorganic bovine-derived bone matrix (ABM) particles embedded in a collagen matrix (henceforth, P-15). The control treatment consisted of PEEK bone dowels with the graft window left void. The P-15 treatment consisted of PEEK bone dowels with P-15 bone graft pellets placed in the bone graft window. Animals were randomly assigned to either 4- or 8-week euthanasia cohorts. Animals in each cohort were then randomly assigned matched-pair treatment to femurs and randomly assigned to undergo either micro-computed tomography (microCT) analysis to investigate bone growth or cytokine analysis to characterize the local immune environment (Figure 2, Table 1).

### 2.3. Surgical Procedures

The surgical procedures performed were similar to those described in published studies evaluating the efficacy of PEEK as an implant material [8,18]. The incision site was identified using fluoroscopy and was made over the lateral aspect of the femur to expose the distal femoral condyle. Osteotomies were performed using a bone drill (Midas Rex, Medtronic) with a series of increasing diameter drill bits at 60,000 RPM. First, a 3 mm matchstick drill bit was used to create a bilateral defect. A 5 mm coarse diamond drill bit was then used to enlarge the defect site to 6 mm to accommodate the implant. The drilling procedure created a critical size bone defect, for which treatment via implant was optimal. During all drilling procedures, 0.9% physiological saline was used to control heat generation and prevent damage to the surrounding bone. Implants were prepared by filling the graft window with the appropriate graft material according to the randomization scheme provided in Table 1 and press fit into the defect site. Appropriate implant placement was confirmed using X-ray and the wound was closed with two layers of suture. The subdermal layer was closed with 4/0 vicryl and the skin layer was closed with 4/0 mersilk sutures. Once the procedure was completed on the first leg, the animal was flipped, and the procedure was repeated to achieve bilateral defects.

**Figure 3:**
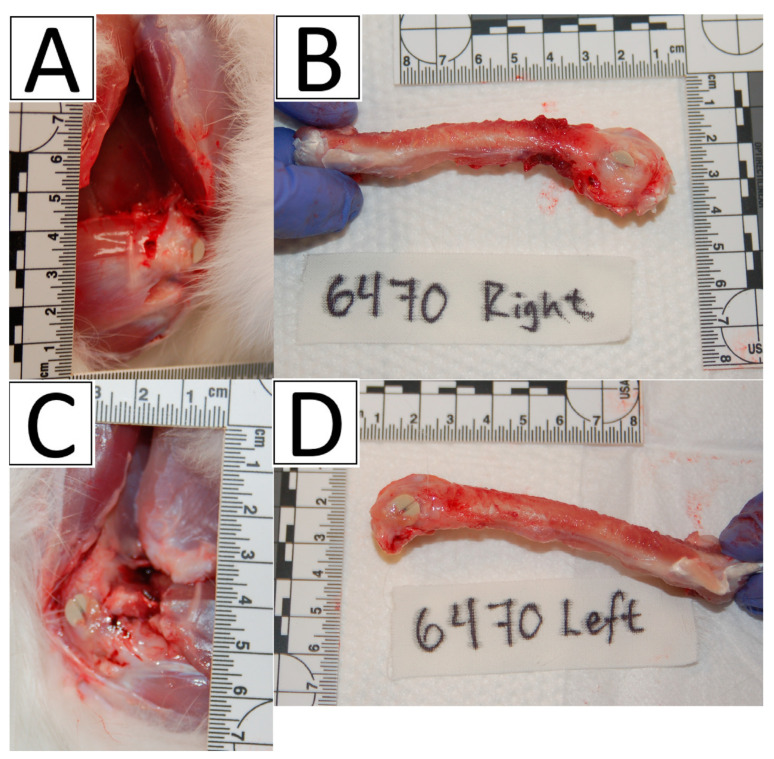
Images illustrating femur collection post-euthanasia and implant placement. (**A**) Sample of right femur osteotomy site. (**B**) Sample of right femur collection. (**C**) Sample of left femur osteotomy site. (**D**) Sample of left femur collection.

At the appropriate euthanasia timepoints, both the left and right femur of each animal were collected (Figure 3). Handling of femur samples was dependent on the type of analysis performed. Samples allocated to the microCT analysis were placed in phosphate buffered formalin. Samples allocated to the cytokine analysis were immediately snap-frozen in liquid nitrogen and transferred to −80 °C storage until cytokine analysis was performed.

### 2.4. MicroCT Analysis

#### 2.4.1. Scanning Procedures

MicroCT scans were performed using a SkyScan 1172 Desktop microCT scanner (Bruker, Billerica, MA, USA). Scans were acquired with an isotropic voxel size of 13.45 µm, aluminum and copper filter, 50 kV, and 200 µA. Each dataset was then reconstructed using NRecon (Bruker) with care taken to ensure that all scans had a consistent linear attenuation to ensure consistency with respect to bone density measurements across samples.

#### 2.4.2. Analysis Procedures

MicroCT analysis was performed using CtAn (Bruker). The analysis was restricted to the central region of each implant to reduce the impact of cortical bone growth and beam hardening artifacts on the analysis. Analysis was conducted within 3 regions of interest (ROIs) surrounding the implant as follows: 1000–500 µm, 500–136 µm, and 136–0 µm normal to the implant surface (Figure 1B). Analysis within each region of interest was achieved using a binary selection threshold which was held constant across all scans.

### 2.5. Cytokine Analysis

**Figure 4:**
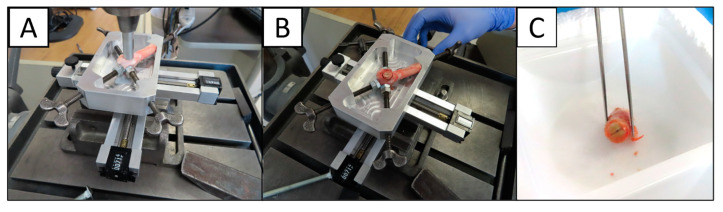
Images illustrating techniques used to collect tissue samples surrounding each implant. (**A**) Coring out the tissue with a hollow drill bit. (**B**) Close-up of method and rig used to hold specimen. (**C**) Cored-out sample.

The bone tissue directly in the graft window or the collar of bone surrounding the implant was removed from the femur using a bone drill with a 10 mm hollow center and drill press (Figure 4). Femurs and all associated tools were sterilized and kept on dry ice during collection. After the implant was removed from the bone, both collar and graft tissue were stored at −80 °C until homogenization.

Samples were homogenized in Tris-HCl using a Fisher-Scientific Bead Mill (Cat. no. 15340164). Tissue homogenates were centrifuged at 4500 RPM for 10 min. The supernatant was collected and centrifuged a second time at 10,000 RPM for 5 min. Total protein concentration of each sample was obtained using the DeNovix Spectrophotometer (DS-11) (DeNovix, Wilmington, DE, USA). Before analysis, samples were all diluted to a working concentration of 2 mg/mL. Cytokine analysis was completed according to manufacturer’s protocols for ELISA kits for IL-2, IL-4, IL-6, TNF-α, and IL-1β (R&D Systems, Minneapolis, MN, USA).

### 2.6. Statistical Analysis

Cytokine levels (pg/mL) in graft and collar samples were analyzed using a mixed-effects model with fixed effects of graft product and timepoint, and with a random effect of rabbit specimen on the intercept. As the tissue cytokine quantification method used in this study was exploratory, *post-hoc* multiple comparisons were performed for significant or marginally significant (*p* < 0.10) effects or interactions, using the estimated marginal means from the model and Tukey’s method of *p* value adjustment. For clarity, interactions are only reported if they had a marginally significant or significant effect. Data from microCT measurements were analyzed for each ROI in an identical fashion (but using the outcome measure of bone volume fraction). Cytokine levels and bone volume fractions are reported as mean (standard deviation). All analyses were performed using R 4.2.1 and the packages *lme4* and *emmeans*. *p* < 0.05 denotes significance.

## 3. Results

### 3.1. Surgical Procedures

From the 17 animals included in this study, the results for 14 animals are included in this analysis (Table 1). The loss of three animals is due to femoral fractures in the post-operative period leading to premature euthanasia. All other animals tolerated the procedure well, with no adverse events reported.

### 3.2. MicroCT Analysis

**Figure 5:**
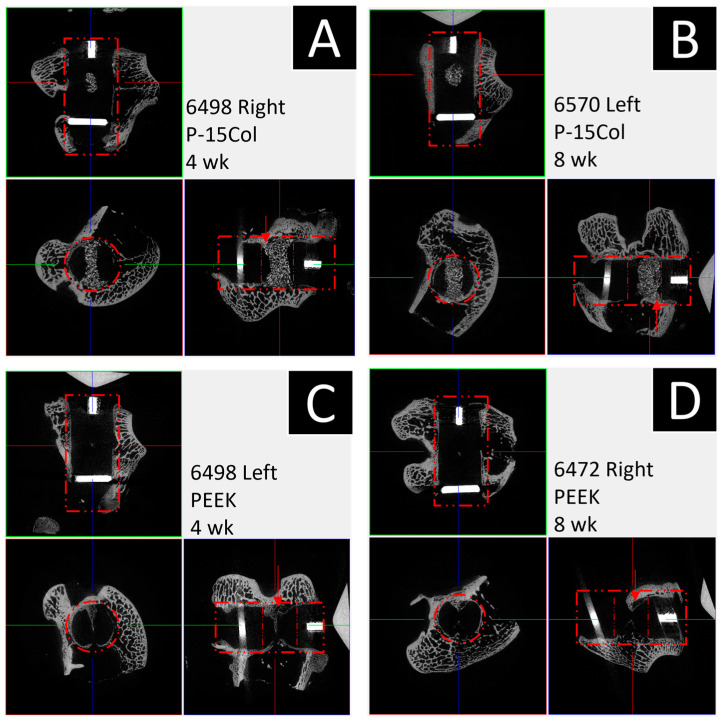
Exemplified microCT scan results for the 8 mm diameter window. Dashed red lines indicate PEEK dowel. Arrows indicate graft window. (**A**) 4-week P-15 cohort; (**B**) 8-week P-15 cohort; (**C**) 4-week polyether-ether-ketone (PEEK) cohort; (**D**) 9-week PEEK cohort. In the P-15 scans, non-absorbed graft material is visible as opaque, higher-density particles.

All microCT scans were performed without issue. Figure 5 below displays examples of scans through the graft window for each of the four cohorts.

**Figure 6:**
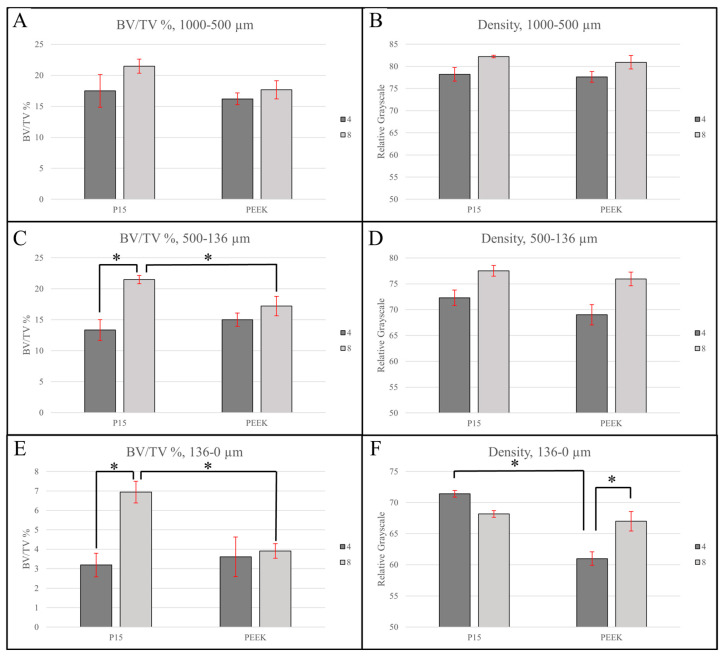
MicroCT analysis results for bone volume fraction and density. (**A**,**B**) 1000–500 µm; (**C**,**D**) 500–136 µm; (**E**,**F**) 136–0 µm ROIs. Significance bars are coded as the following: * = *p* < 0.05.

Figure 6 below includes the bone volume fraction (BV/TV) (Table 2) and bone density results (Table 3) from this analysis. The results of the bone density analysis are reported in relative grayscale values (GVs). Statistical analyses were performed for each ROI as described below.

#### 3.2.1. Large Region of Interest (1000–500 µm)

With respect to BV/TV measurements within the largest ROI, which was 1000–500 µm from the implant surface, the model showed no significant effects of treatment (F(1, 4) = 1.1445, *p* = 0.3449) or timepoint (F(1, 4) = 1.3123, *p* = 0.3159). Similarly, the model showed no significant effects of treatment (F(1, 4) = 0.6041, *p* = 0.4804) or timepoint (F(1, 4) = 2.7495, *p* = 0.1726) with respect to bone density measurements in this ROI.

#### 3.2.2. Intermediate Region of Interest (500–136 µm)

With respect to the second ROI, which was 500–136 µm from the implant surface, the model showed no significant effects of treatment (F(1, 4) = 2.6372, *p* = 0.1797) or timepoint (F(1, 4) = 4.2980, *p* = 0.1068), however, the interaction between these terms was significant (F(1, 4) = 13.6599, *p* = 0.0209) and pairwise differences were, therefore, examined. This showed a significant difference in BV/TV between the PEEK and P-15 cohorts at the 8-week timepoint (*p* = 0.0197) as well as a significant difference between BV/TV observed for the P-15 cohort at 4 weeks and 8 weeks (*p* = 0.0280). With respect to density measurements in the 500–136 µm ROI, the model showed no significant effects of treatment (F(1, 4) = 4.0636, *p* = 0.1140) or timepoint (F(1, 4) = 4.8809, *p* = 0.0917), however, given the exploratory nature of this study, the main effect of timepoint was considered marginally significant and pairwise comparisons were, therefore, evaluated. These comparisons showed a trend of increasing bone density between the bone density in the PEEK cohort observed at 4 and 8 weeks (*p* = 0.0643).

#### 3.2.3. Small Region of Interest (136–0 µm)

With respect to the smallest ROI, which was 136–0 µm from the implant surface, the model showed no significant effects of treatment (F(1, 4) = 2.9491, *p* = 0.1611) or timepoint (F(1, 4) = 3.2411, *p* = 0.1462), however, the interaction between these terms was considered marginally significant (F(1, 4) = 5.2106, *p* = 0.0845) and due to the exploratory nature of this study, pairwise comparisons were, therefore, evaluated. These comparisons indicated a significant difference in the BV/TV measurement of PEEK and P-15 at the 8-week timepoint (*p* = 0.047), as well as a significant difference between BV/TV observed for the P-15 cohort at 4 and 8 weeks (*p* = 0.0277). The investigation of bone density measurements within this smallest ROI showed the main effect of treatment had a significant effect (F(1, 4) = 23.1464, *p* = 0.0086) while the effect of timepoint was insignificant (F(1, 4) = 0.7003, *p* = 0.4498), however, the interaction term between the treatment cohort and timepoint was significant (F(1, 4) = 14.7768, *p* = 0.0184) and all pairwise comparisons were, therefore, examined. These pairwise comparisons showed a significant difference between the PEEK and P-15 cohorts at the 4-week timepoint (*p* = 0.0036) as well as a significant difference in bone density between the PEEK cohorts at 4 and 8 weeks (*p* = 0.0209).

### 3.3. Cytokine Analysis

**Figure 7:**
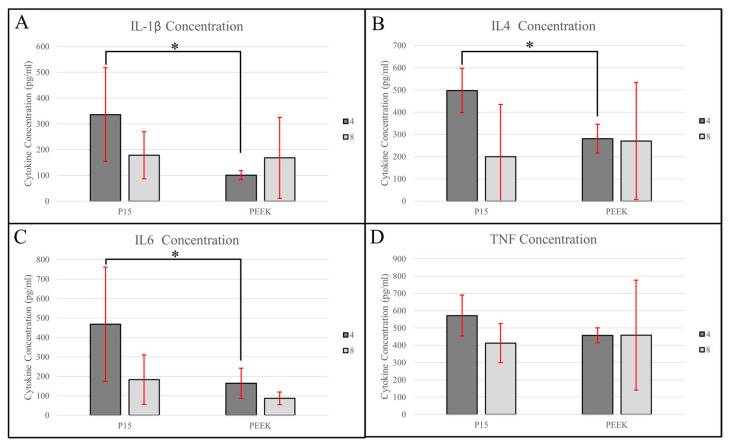
Cytokine analysis results for (**A**) IL-1β; (**B**) IL-4; (**C**) IL-6; and (**D**) TNF-α. IL-2 was excluded due to sample sizes. Significance bars are coded as the following: * = *p* < 0.05.

ELISAs were performed on tissue samples taken from the periprosthetic region, which was 2 mm normal to the implant surface (Figure 7, Table 4). A total of four samples were analyzed from each cohort. There were two PEEK samples from the 4-week cohort and three PEEK samples from the 8-week cohort that had undetectable levels of IL-2, therefore, statistical analysis was not performed relative to IL-2 due to the small and uneven sample sizes.

#### 3.3.1. IL-1β

With respect to IL-1β concentration, the model showed no significant effects of treatment (F(1, 6) = 5.0293, *p* = 0.0661) or timepoint (F(1, 6) = 0.3893, *p* = 0.5556). Since the main effect of treatment was considered marginally significant, pairwise comparisons were evaluated. This showed a significant difference between the IL-1β concentration between the PEEK and P-15 cohorts at the 4-week timepoint (*p* = 0.0228).

#### 3.3.2. IL-4

With respect to IL-4 concentration, the model showed that the main effect of the treatment group had a significant effect (F(1, 6) = 8.5076, *p* = 0.0267) and the effect of timepoint had an insignificant effect (F(1, 6) = 1.4278, *p* = 0.2772), however, the interaction term between the treatment group and timepoint was significant (F(1, 6) = 32.7333, *p* = 0.0012) and, therefore, all pairwise comparisons were evaluated. These comparisons showed a significant difference between the IL-4 concentration observed between the PEEK and P-15 cohorts at 4 weeks (*p* = 0.0009). The level of IL-4 may have been lower in the P-15 cohort at 8 weeks as compared to 4 weeks, but this difference was not significant (*p* = 0.0612).

#### 3.3.3. IL-6

With respect to IL-6 concentration, the model showed the main effects of the treatment group (F(1, 6) = 5.8330, *p* = 0.0522) and timepoint (F(1, 6) = 4.7747, *p* = 0.0716) were both marginally significant. Therefore, all pairwise comparisons were evaluated, showing a significant difference between the IL-6 concentration between the PEEK and P-15 cohorts at the 4-week timepoint (*p* = 0.0410) as well as a significant difference between the IL-6 concentrations within the P-15 cohorts at 4 and 8 weeks (*p* = 0.0317).

#### 3.3.4. TNF-α

Neither treatment (F(1, 6) = 0.2498, *p* = 0.6350) nor timepoint (F(1, 6) = 0.5386, *p* = 0.4970) had a significant effect on TNF-α concentration.

## 4. Discussion

Modulating the immune response to promote bony deposition is an important endpoint when designing implants from biomaterials for applications involving bone healing. P-15 peptide was shown to alter the response to PEEK measured in this study by both immunomodulation and mineralization. Specifically, we observed improved bone growth in the periprosthetic region, and a more active micro-environment as indicated by cytokine concentrations in the regions directly surrounding the implant as compared to PEEK alone.

The microCT analysis performed was intended to investigate the influence of P-15 on the surrounding tissues while minimizing imaging artifacts and the influence of residual graft products. Analysis was, therefore, performed in ROIs surrounding the implant, with the central graft window excluded from analysis. Other authors have used methods like these to assess bone growth surrounding bone dowels and reported BV/TV results surrounding PEEK implants that are similar [19,20]. Using ROIs with increasing distance from the implant surface allowed for the investigation of how far the P-15 zone of influence extends into the local environment. The size of the smallest ROI was chosen to focus on bone growth only within the periprosthetic region and is correlated with the thickness of fibrous capsule formation [21]. ROIs closer to the implant surface demonstrated significant bone formation in this experiment. Both the 500–136 and 136–0 µm ROIs showed a significantly higher bone volume fraction in the P-15 cohort as compared to the PEEK cohort.

Not only was more bone observed in this region for the P-15 cohort, but the bone had a higher density, indicating a more mature bony environment. Statistically significant temporal changes in bone volume fraction were also observed, however, these 4- to 8-week differences were only observed in the P-15 cohort. Taken together, these results indicate that P-15 supports faster bone deposition and transition from immature woven bone to mature mineralized tissues. This is in agreement with studies suggesting the treatment of PEEK with osteointegrative substances can increase the volume of bone growth compared to PEEK alone [7]. Furthermore, this potentially suggests that treatment with PEEK and P-15 could yield lower rates of pseudoarthrosis compared to treatment with PEEK alone [12].

The trends in cytokine expression suggest that P-15 samples had a more active cellular environment, as indicated by the comparatively higher cytokine expression for most cytokines and timepoints. The concentration of each of the four cytokines evaluated remained unchanged from four to eight weeks in the control group, whereas significant and near-significant temporal changes were observed in the cohort treated with P-15. We hypothesize this change in cytokine expression may indicate a shift from the repair to remodeling phase between 4 and 8 weeks [2]. This contrasts strongly with the prolonged pro-inflammatory state reported in the literature [8,9].

TNF-α is a pluripotent cytokine which plays an important role in both early bone healing as well as the remodeling stage. Early expression of TNF-α is thought to come from resident macrophages and plays a role in osteoblast differentiation [22]. TNF-α is upregulated in the remodeling stage of healing and functions to promote osteoclastogenesis [23]. The persistent and elevated expression of TNF-α levels in tissue can cause damage and reduce bone volume [24]. There was no statistically significant effect of time or treatment group on the TNF-α found, however, a potential decrease from the 4-week to 8-week timepoint is observed in the P-15 group, while the average value for PEEK showed no change. Additionally, the P-15 group at 4 weeks had higher levels than the control (although, this difference was not statistically significant). Taken together, these results may provide positive evidence that the second peak of pro-inflammatory cytokines at the remodeling stage of healing was reached at an earlier stage in the presence of P-15, while the control group was either undergoing the secondary pro-inflammatory peak or was experiencing detrimental sustained inflammatory cytokine expression.

A second inflammatory cytokine involved in late-stage remodeling is IL-1β. The data suggest a potential decrease in IL-1β concentration from 4 to 8 weeks in the P-15 cohort. In fracture models, IL-1β is produced by osteoblasts at three weeks post-injury to stimulate bone remodeling [25]. Here, there was a significant difference in the IL-1β concentration between the control and P-15 at 4 weeks. This may indicate that osteoblasts and other cell types increase the concentration of IL-1β in the bone tissue when P-15 is present, as compared to the control. The presence of the ABM in the P-15 product used in this study may be another reason for the elevated IL-1β levels relative to the PEEK cohort. IL-1β is known to stimulate bone resorption, both directly through its influence on osteoclastic activity and indirectly through the RANKL pathway, which drives osteoclastogenesis [26].

IL-6 is a pro-inflammatory cytokine that is upregulated in the early inflammatory phase of healing in response to IL-1β stimulation [16]. It is also a crucial regulator of the remodeling process through the cartilage-to-bone transformation and bony callus remodeling [27]. The data show a significant decrease in IL-6 expression from 4 to 8 weeks in the P-15 cohort, as well as significantly lower levels of IL-6 in the 4-week PEEK cohort relative to P-15. These results would suggest greater levels of IL-6 action towards successful bone remodeling in the presence of P-15, and that P-15 does not cause sustained levels of this pro-inflammatory cytokine.

In bone healing, an increase in anti-inflammatory cytokines is necessary to shift from the inflammatory to repair phase and reduce the risk of chronic inflammation. This is of particular importance to the consideration of PEEK as an implant material due to its increased inflammatory response and resulting periprosthetic formation of fibrotic and adipose tissue [7,8,9]. We observed significantly higher IL-4 levels in the P-15 cohort at 4 weeks compared to the PEEK control. Increased IL-4 expression may contribute to macrophage polarization towards the M2 phenotype. These alternatively activated macrophage immunoregulatory properties and helped to facilitate tissue repair through the expression of IL-10 and TGF-β [28,29,30]. The increased expression of IL-10 has also been reported in studies investigating the immune response to PEEK [9]. Additionally, the IL-4 level appeared to decrease from 4 to 8 weeks in the P-15 cohort (a large, but non-significant difference), which could further support the hypothesized progression from the repair to remodeling phase. Future analysis would benefit from looking at IL-4 expression 1–2 weeks post-implantation to better understand the modulation of expression.

This study has several limitations. The rabbit distal femur defect model utilized here does not perfectly represent bone healing in humans. For example, the remodeling of cortical bone in young rabbits occurs at a relatively high rate and may not reflect the remodeling of human bone [31]. However, this model does allow for a relative comparison of bone formation and immune response in the presence and absence of P-15 bone graft (using a distal femur defect model treated with a PEEK implant). Another important limitation is that bone volume fraction, bone density, and cytokine levels were measured at just two post-operative timepoints, and that cytokine levels could not be assessed at baseline. Cytokine levels must be examined in a more continuous fashion due to natural variability with time. While the sample size was small, the study provides a preliminary understanding of the magnitude of change in immune response with P-15 as a bone graft material. Thus, the study can be used to power and design further studies that can better explore the immunological responses. Nevertheless, the data presented here provide preliminary evidence of the effect of the presence of P-15 bone graft on bone formation and inflammation at two post-operative timepoints. The findings presented here can be used to inform the design of further studies evaluating P-15 as a bone graft material, including large-animal studies and clinical trials.

## 5. Conclusions

This study evaluated bone formation and immune response following the application of P-15 as a bone graft material using a rabbit femoral defect model treated with a PEEK implant. Greater bone formation was observed in ROIs close to the implant surface in rabbits treated with P-15. Cell surface interaction with biomaterials induces intracellular signaling cascades, which dictate cell differentiation, proliferation, and extracellular signaling. The levels of pro-inflammatory cytokines (TNF-α, IL-1β, and IL-6) as well as an anti-inflammatory cytokine (IL-4) at four- and eight-weeks post-implantation measured in this preliminary study suggest that those treated with P-15 may have undergone an accelerated transition to the remodeling period. Larger studies with a more continuous examination of changes in cytokine levels are needed to further understand how P-15 affects the immune response to injury, implant, and bone graft material, and how these changes relate to enhanced bone formation.

## Figures and Tables

**Table 1:** Randomization of animals to timepoint and treatment cohorts (N/A indicates animals euthanized early and not include in analysis).

Animal	Cohort	Left	Right	Analysis
1	8	P-15	Control	MicroCT
2	8	Control	P-15	Cytokine
3	8	P-15	Control	MicroCT
4	8	Control	P-15	Cytokine
5	8	P-15	Control	MicroCT
6	N/A	Control	P-15	Cytokine
7	N/A	P-15	Control	Cytokine
8	N/A	Control	P-15	Cytokine
9	8	P-15	Control	Cytokine
10	8	Control	P-15	Cytokine
11	4	Control	P-15	MicroCT
12	4	Control	P-15	Cytokine
13	4	P-15	Control	MicroCT
14	4	P-15	Control	Cytokine
15	4	Control	P-15	MicroCT
16	4	Control	P-15	Cytokine
17	4	P-15	Control	Cytokine

**Table 2:** Bone volume fraction by cohort and timepoint.

Region of Interest	Timepoint (Week)	Mean Bone Volume Fraction (%)		Adjusted Mean Bone Volume Fraction (%) with 95% CI *		Adjusted *p* Value **^,†^			
						PEEK and P-15 at Week 4	PEEK and P-15 at Week 8	PEEK from Week 4 to 8	P-15 from Week 4 to 8
		PEEK	P-15	PEEK	P-15				
Large (1000–500 µm)	4 (*n* = 3)	16.2 ± 2.3	17.5 ± 6.4	16.2 ± 2.4 (10.7–21.7)	17.5 ± 2.4 (12.0–23.0)	N/A	N/A	N/A	N/A
	8 (*n* = 3)	17.7 ± 3.6	21.5 ± 2.8	17.7 ± 2.4 (12.2–23.1)	21.5 ± 2.4 (16.0–26.9)				
Intermediate (500–136 µm)	4 (*n* = 3)	15.0 ± 2.6	13.3 ± 4.1	15.0 ± 1.9 (10.2–19.8)	13.3 ± 1.9 (8.5–18.2)	0.22	0.020	0.44	0.028
	8 (*n* = 3)	17.2 ± 3.9	21.5 ± 1.6	17.2 ± 1.9 (12.4–22.0)	21.5 ± 1.9 (16.7–26.3)				
Small (136–0 µm)	4 (*n* = 3)	3.6 ± 2.5	3.2 ± 1.5	3.6 ± 1.0 (1.4–5.9)	3.2 ± 1.0 (0.9–5.5)	0.71	0.047	0.83	0.028
	8 (*n* = 3)	3.9 ± 0.9	6.9 ± 1.4	3.9 ± 1.0 (1.6–6.2)	6.9 ± 1.0 (4.7–9.2)				

Descriptive means are presented as mean ± standard deviation. Adjusted means were obtained from the mixed model analysis and are presented as estimate ± standard error. Adjusted *p* < 0.05 denotes significance. * Confidence interval; ** adjusted for multiple comparisons; ^†^ N/A indicates the test was not performed as the main effect or interaction was not at least marginally significant.

**Table 3:** Bone density by cohort and timepoint.

Region of Interest	Timepoint (Week)	Mean Density (GV *)		Adjusted Mean Density (GV) with 95% CI **		Adjusted *p* Value ^†,††^			
						PEEK and P-15 at Week 4	PEEK and P-15 at Week 8	PEEK from Week 4 to 8	P-15 from Week 4 to 8
		PEEK	P-15	PEEK	P-15				
Large (1000–500 µm)	4 (*n* = 3)	77.6 ± 3.0	78.2 ± 3.8	77.6 ± 1.8 (73.3–81.9)	78.2 ± 1.8 (73.9–82.5)	N/A	N/A	N/A	N/A
	8 (*n* = 3)	80.9 ± 3.7	82.2 ± 0.7	80.9 ± 1.8 (76.6–85.2)	82.2 ± 1.8 (77.9–86.5)				
Intermediate (500–136 µm)	4 (*n* = 3)	69.0 ± 4.8	72.3 ± 3.7	69.0 ± 2.1 (63.7–74.3)	72.3 ± 2.1 (67.0–77.6)	N/A	N/A	0.064	0.14
	8 (*n* = 3)	75.9 ± 3.3	77.5 ± 2.5	75.9 ± 2.1 (70.6–81.2)	77.5 ± 2.1 (72.2–82.8)				
Small (136–0 µm)	4 (*n* = 3)	61.0 ± 2.7	71.4 ± 1.3	61.0 ± 1.5 (57.6–64.4)	71.4 ± 1.5 (68.0–74.8)	0.0036	0.53	0.021	0.16
	8 (*n* = 3)	67.0 ± 3.8	68.2 ± 1.3	67.0 ± 1.5 (63.6–70.4)	68.2 ± 1.5 (64.8–71.6)				

Descriptive means are presented as mean ± standard deviation. Adjusted means were obtained from the mixed model analysis and are presented as estimate ± standard error. Adjusted *p* < 0.05 denotes significance. * Relative grayscale value; ** confidence interval; ^†^ adjusted for multiple comparisons; ^††^ N/A indicates the test was not performed as the main effect or interaction was not at least marginally significant.

**Table 4:** Cytokine levels by cohort and timepoint.

Cytokine	Timepoint (Week)	Mean Cytokine Level (pg/mL)		Adjusted Mean Cytokine Level (pg/mL) with 95% CI *		Adjusted *p* Value **^,†^			
						PEEK and P-15 at Week 4	PEEK and P-15 at Week 8	PEEK from Week 4 to 8	P-15 from Week 4 to 8
		PEEK	P-15	PEEK	P-15				
IL-1β	4 (*n* = 4)	101.1 ± 17.6	336.2 ± 182.3	101.0 ± 64.5 (−40.8–243.0)	336.0 ± 64.5 (194.3–478.0)	0.023	0.90	0.48	0.11
	8 (*n* = 4)	167.9 ± 157.4	178.1 ± 91.3	168.0 ± 64.5 (26.1–310.0)	178.0 ± 64.5 (36.3–320.0)				
IL-4	4 (*n* = 4)	281.2 ± 64.6	497.5 ± 100.0	281.0 ± 92.9 (57.7–505.0)	497.0 ± 92.9 (274.0–721.0)	0.0009	0.095	0.94	0.061
	8 (*n* = 4)	270.4 ± 262.4	200.1 ± 234.6	270.0 ± 92.9 (46.8–494.0)	200.0 ± 92.9 (−23.4–424.0)				
IL-6	4 (*n* = 4)	164.2 ± 78.0	467.6 ± 293.9	164.0 ± 82.8 (−16.1–344.0)	468.0 ± 82.8 (287.3–648.0)	0.041	0.44	0.52	0.032
	8 (*n* = 4)	87.0 ± 32.8	183.2 ± 126.6	87.0 ± 82.8 (−93.3–267.0)	183.0 ± 82.8 (2.9–364.0)				
TNF-α	4 (*n* = 4)	457.5 ± 43.5	571.6 ± 118.6	458.0 ± 90.1 (257.0–658.0)	572.0 ± 90.1 (371.0–772.0)	N/A	N/A	N/A	N/A
	8 (*n* = 4)	458.6 ± 317.8	412.3 ± 113.3	459.0 ± 90.1 (258.0–659.0)	412.0 ± 90.1 (212.0–613.0)				

Descriptive means are presented as mean ± standard deviation. Adjusted means were obtained from the mixed model analysis and are presented as estimate ± standard error. Adjusted *p* < 0.05 denotes significance. * Confidence interval; ** adjusted for multiple comparisons; ^†^ N/A indicates the test was not performed as the main effect or interaction was not at least marginally significant.

## Data Availability

The original contributions presented in the study are included in the article, further inquiries can be directed to the corresponding author.
